# A Review on the Biotechnological Applications of the Operational Group *Bacillus amyloliquefaciens*

**DOI:** 10.3390/microorganisms9030614

**Published:** 2021-03-17

**Authors:** Mohamad Syazwan Ngalimat, Radin Shafierul Radin Yahaya, Mohamad Malik Al-adil Baharudin, Syafiqah Mohd. Yaminudin, Murni Karim, Siti Aqlima Ahmad, Suriana Sabri

**Affiliations:** 1 Enzyme and Microbial Technology Research Center, Faculty of Biotechnology and Biomolecular Sciences, Universiti Putra Malaysia, Serdang 43400, Selangor, Malaysia; syazwanngalimat@gmail.com (M.S.N.); radinshafierul@yahoo.com (R.S.R.Y.); malikaladilbaharudin@gmail.com (M.M.A.-a.B.); 2 Department of Aquaculture, Faculty of Agriculture, Universiti Putra Malaysia, Serdang 43400, Selangor, Malaysia; nsmy211195@gmail.com (S.M.Y.); murnimarlina@upm.edu.my (M.K.); 3 Laboratory of Sustainable Aquaculture, International Institute of Aquaculture and Aquatic Sciences, Universiti Putra Malaysia, Port Dickson 71050, Negeri Sembilan, Malaysia; 4 Department of Biochemistry, Faculty of Biotechnology and Biomolecular Sciences, Universiti Putra Malaysia, Serdang 43400, Selangor, Malaysia; aqlima@upm.edu.my; 5 Department of Microbiology, Faculty of Biotechnology and Biomolecular Sciences, Universiti Putra Malaysia, Serdang 43400, Selangor, Malaysia

**Keywords:** plant growth-promoting bacteria, biocontrol agent, enzymes, antimicrobial compounds, probiotics, bioremediation, *Bacillus amyloliquefaciens*, *Bacillus velezensis*, *Bacillus siamensis*, *Bacillus nakamurai*

## Abstract

Bacteria under the operational group *Bacillus amyloliquefaciens* (OG*Ba*) are all Gram-positive, endospore-forming, and rod-shaped. Taxonomically, the OG*Ba* belongs to the *Bacillus subtilis* species complex, family Bacillaceae, class Bacilli, and phylum Firmicutes. To date, the OG*Ba* comprises four bacterial species: *Bacillus amyloliquefaciens*, *Bacillus siamensis*, *Bacillus velezensis* and *Bacillus nakamurai*. They are widely distributed in various niches including soil, plants, food, and water. A resurgence in genome mining has caused an increased focus on the biotechnological applications of bacterial species belonging to the OG*Ba*. The members of OG*Ba* are known as plant growth-promoting bacteria (PGPB) due to their abilities to fix nitrogen, solubilize phosphate, and produce siderophore and phytohormones, as well as antimicrobial compounds. Moreover, they are also reported to produce various enzymes including α-amylase, protease, lipase, cellulase, xylanase, pectinase, aminotransferase, barnase, peroxidase, and laccase. Antimicrobial compounds that able to inhibit the growth of pathogens including non-ribosomal peptides and polyketides are also produced by these bacteria. Within the OG*Ba*, various *B. velezensis* strains are promising for use as probiotics for animals and fishes. Genome mining has revealed the potential applications of members of OG*Ba* for removing organophosphorus (OPs) pesticides. Thus, this review focused on the applicability of members of OG*Ba* as plant growth promoters, biocontrol agents, probiotics, bioremediation agents, as well as producers of commercial enzymes and antibiotics. Here, the bioformulations and commercial products available based on these bacteria are also highlighted. This review will better facilitate understandings of members of OG*Ba* and their biotechnological applications.

## 1. Introduction

In 1943, a Japanese scientist, Juichiro Fukumoto, first isolated *Bacillus amyloliquefaciens* from the soil. The species is named after its unique character because it produced (*faciens*) a liquefying (*lique*) *α*-amylase (*amylo*) [1,2]. Later, *B. amyloliquefaciens* was combined with the closely related *Bacillus subtilis* and *Bacillus licheniformis* into the *B. subtilis* species complex, based on phylogenetic and phenetic evidence [3]. From the *B. subtilis* species complex, it can be further sub-grouped into the operational group *B. amyloliquefaciens* (OG*Ba*) that comprises four bacterial species; the soil-borne *B. amyloliquefaciens*, the plant-associated *Bacillus siamensis* and *Bacillus velezensis*, and a black-pigment-producing strain *Bacillus nakamurai* [4].

Previously, several bacterial species of the OG*Ba*, namely *B. amyloliquefaciens* subsp. *plantarum*, *Bacillus methylotrophicus* and *Bacillus oryzicola,* were reclassified as strains of *B. velezensis* [5]. Genome-based and gene-derived phylogenetic analyses revealed that *B. velezensis* belongs to a conspecific group consisting of *B. velezensis*, *B. amyloliquefaciens* subsp. *plantarum* FZB42 (reclassified as *B. velezensis* FZB42) and *B. methylotrophicus*. However, *B. velezensis* is distinct from the closely related species of *B. amyloliquefaciens* and *B. siamensis* [4]. To date, a plethora of bacterial whole-genome sequences (WGS) from members of OG*Ba* have been deposited into the National Center Biological Information (NCBI) database (Table S1). As confirmed taxonomically in 2019, 223 genomes belonged to *B. velezensis*, 19 belonged to *B. amyloliquefaciens*, 10 belonged to *B. siamensis* and 2 belonged to *B. nakamurai* [6].

The members of OG*Ba* are found in various niches including soil, plants, food, animal faeces and aquatic environments [4]. Currently, genome mining has revealed their applicability as plant growth-promoters, biocontrol agents, probiotics, bioremediation agents as well as producers of commercial enzymes and antibiotics [7,8]. Therefore, knowledge of the biology of the OG*Ba* is imperative to understanding the special qualities of the group. This review focused on the biotechnological applications of the bacterial strains belonging to the OG*Ba*.

## 2. An Overview of the OG*Ba*

### 2.1. Identification and Characterization

Bacterial species from the OG*Ba* are all Gram-positive bacteria and motile by peritrichous flagella. They are endospore-forming bacteria from the *B. subtilis* species complex. For many years, the speciation of OG*Ba* within the *B. subtilis* species complex has been uncertain, often leading to erroneous and variable results. They are difficult to distinguish using classical taxonomy parameters: morphological and physiological characteristics, cell wall compositions, 16S ribosomal RNA sequence, guanine–cytosine (G+C) content, fatty acid methyl esters (FAME) and DNA–DNA hybridization (DDH) [9]. Therefore, the taxonomic status of the bacterial species belonging to the OG*Ba* is constantly causing confusion to researchers, especially for non-professional taxonomy researchers.

**Figure 1:**
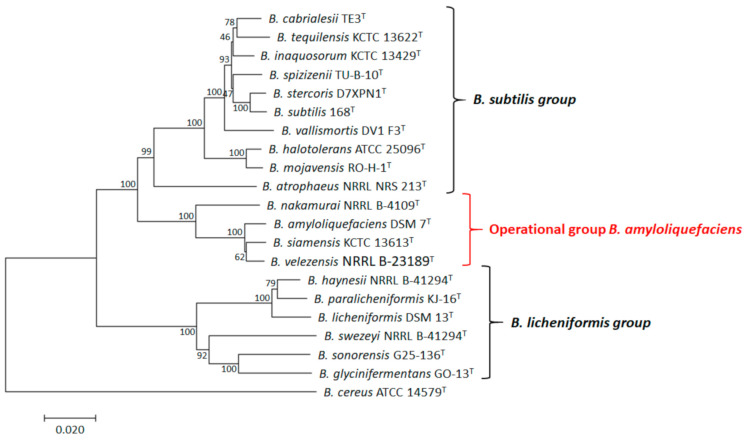
Neighbor-joining phylogenetic tree based on complete *rpoB* nucleotide sequences of bacterial species under the *B. subtilis* species complex. Evolutionary analyses were conducted using the MEGAX software [15]. The optimal tree with the sum of branch length = 0.66533958 is shown. The evolutionary distances were computed using the *p*-distance method. Bootstrap values, based on 1000 repetitions, are indicated at the branch points. The analysis involved 19 nucleotide sequences. There were 3534 positions in the final dataset. Bar, 0.02 substitutions per nucleotide position. *Bacillus cereus* ATTC 14579^T^ was used as the outgroup.

It is worth mentioning that some studies have used protein-coding genes in order to further ascertain the degree of relatedness of the OG*Ba* within the *B. subtilis* species complex [10,11]. The highly conserved DNA gyrase subunit B (*gyrB*), signal transduction histidine kinase CheA (*cheA*) and RNA polymerase *β*-subunit (*rpoB*) were used for the study of speciation within the *B. subtilis* species complex before the advent of multilocus sequence analysis (MLSA) [11,12,13]. The taxonomical status of the members of OG*Ba* has been solved by genome-based [4] and gene-derived [14] phylogeny analyses. The OG*Ba* comprised four species: (i) *B. amyloliquefaciens*; (ii) *B. siamensis*; (iii) *B. velenzensis*; and (iv) *B. nakamurai*, as confirmed by cladistic analysis (Figure 1; Table 1).

### 2.2. Ecology, Isolation and Cultivation

The ability to produce endospores when facing harsh conditions allowed the members of the operational group to survive in various niches including soil, animal faeces, plants, food, bee products, drugs, air, and the aquatic environments (Table S1). Evidently, the members of OG*Ba* had been directly isolated from rare dormant volcanic soils [19], mango orchards [20] and animal faeces [21,22]. They had also been isolated from plant parts including fruits (such as lemons [23] and apples [24]), roots (such as Peruvian ground cherry [25] and peanut roots [26]) and leaves (such as lucerne [27] and camphor leaves [28]).

Moreover, traditional fermented foods including bibimbap [29], douchi [30], and doenjang [31] were reported as the sources of isolation of bacteria from this operational group. They also were isolated from bee products [32,33,34], heroin [35], and air [36]. In other related studies, bacteria of this operational group have been isolated from water [37], seawater [38] and sea sediment [39]. Chicken [40] and fish intestines [41] were also reported as the sources of origin for members of this operational group.

Generally, the members of OG*Ba* are cultivated routinely in Luria–Bertani (LB) medium at 30–37 °C aerobically [11,16,17]. Some members of OG*Ba* such as *B. nakamurai* grew well on nutrient agar (NA), trypticase soy agar (TSA), Reasoner’s 2A agar (R2A) and tryptone glucose yeast extract agar (TGY) at 30 °C for two days [18]. Moreover, *B. velezensis* and *B. siamensis* were also reported to grow well on TSA at 37 °C and 32 °C, respectively [16,17].

### 2.3. Genome and Its Arrangement

In 2019, 254 bacterial strain genomes which had been deposited in the NCBI database were reported as belonging to the OG*Ba* [6]. Some of the examined strains were found to contain plasmids (Table S1). Most of the reported strains had only one plasmid, except for *B. velezensis* 157, *B. velezensis* DKU_NT_04, and *B. velezensis* NJAU-Z9 (all contained two plasmids), and *B. velezensis* LB002 (which contained three plasmids). Interestingly, some studies have focused on the functionality of the genes carried by the plasmid. For instance, the *B. velezensis* S499 plasmid, pS499, was reported as containing a *rap-phr* cassette. This cassette encoded for the regulator aspartate phosphatase (*rap*) and the Rap regulatory peptide (*phr*) with a role in governing protease secretion, growth and motility, biofilm formation and production of surfactin [42]. Meanwhile, *B. amyloliquefaciens* LL3 plasmid, pMC1, has a 6.8 kbp plasmid that includes a *rap* which is not homologous to the pS499 [42]. The hypothetical *rap* and the origin of replication of the pMC1 plasmid were cloned into the pKSV7, vector which brought about the production of plasmid-cured strains. The plasmid-cured strains have increases in glutamate-independent poly-*γ*-glutamic acid production by 6% as compared to the *B. amyloliquefaciens* LL3 [43].

Genome analysis allowed for further biological studies on the members of OG*Ba*. The genomic and metabolic features of the members of the group were similar; however, species-specific features including secondary metabolite biosynthesis-related and energy metabolism-related genes were also identified [4,44]. Secondary metabolite biosynthesis-related genes are enriched in *B. velezensis*, whereas energy metabolism-related genes are enriched in *B. amyloliquefaciens*. In the core-genome, *B. velezensis* harbors more genes involved in the biosynthesis of antimicrobial compounds as well as genes involved in _D_-galacturonate and _D_-fructuronate metabolisms compared to *B. amyloliquefaciens* and *B. siamensis*. Moreover, a xanthine oxidase gene cluster that is involved in metabolizing xanthine and uric acid to glycine and oxalureate was found in the core-genome of all the members of the group. Pan-genome analysis revealed the abilities of members of OG*Ba* to metabolize diverse carbon sources aerobically or anaerobically. Their abilities to produce various metabolites such as lactate, ethanol, xylitol, diacetyl, acetoin, and 2,3-butanediol were also identified [44]. In addition, genome analysis suggested that the regions of genomic plasticity controlled the function and structure of the genome and governed the adaptations to different niches [45]. Genome analysis also enabled the prediction of uncharacterized gene clusters and assessed the capabilities of members of OG*Ba* to produce antimicrobial compounds [6].

## 3. The Importance and Applications of the OG*Ba*

### 3.1. Plant Growth Promoters and Biocontrol Agents in Agriculture

**Figure 2:**
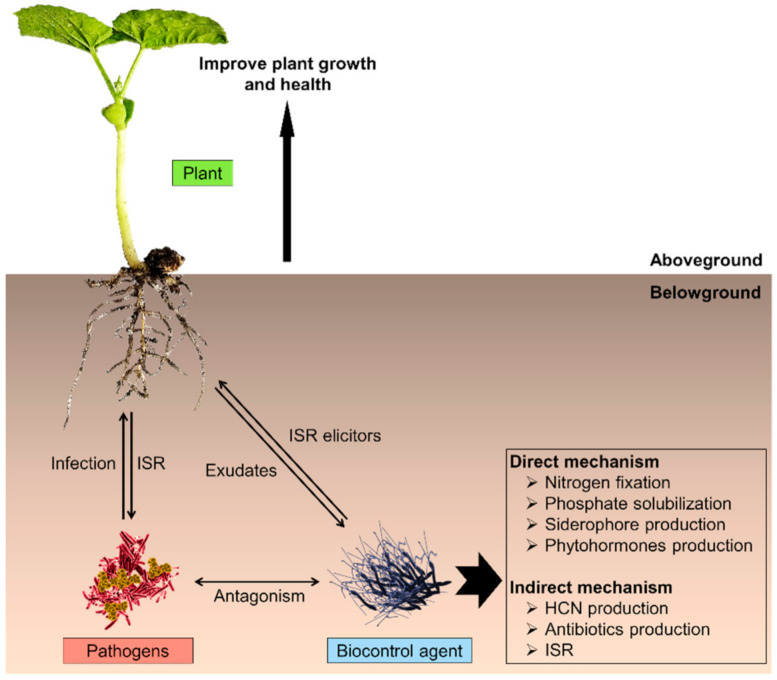
The biological control interactions. The illustration depicts the interactions between biocontrol agents, plant pathogens, and plants. The biocontrol agent colonized the plant root surface and produced antimicrobial compounds such as surfactin. In the plant rhizosphere, antibiosis and nutrient competition interaction suppressed the growth of pathogens. Due to the production of antimicrobial compounds and in the simultaneous presence of pathogens, the induced systemic resistance (ISR) is enhanced. Thus, this mediated the defense response of the plant towards pathogens and consequently improved plant growth and the defense mechanism against pathogens.

In the agricultural sector, the biocontrol strategy has received great attention because it provides safe, environmentally friendly, long-lasting, and inexpensive alternatives [46]. The characterizations of the bacterial strains from the OG*Ba* as biocontrol agents were determined based on their abilities to improve plant growth and health [47]. These abilities involve multiple mechanisms including direct (improve plant growth) and indirect (improve plant health) mechanisms (Figure 2). Direct mechanisms involve nitrogen fixation, phosphate solubilization, siderophore production and phytohormone production (e.g., indole-3-acetic acid (IAA) and enzymes such as 1-amyclocyclopropane-1-carboxylate (ACC) deaminase). It has been reported that the co-inoculation of *B. velezensis* S141 with *Bradyrhizobium diazoefficiens* USDA110 into soybean resulted in enhanced nodulation and nitrogen fixation efficiency by producing larger nodules [48]. In another related study, the members of OG*Ba* were able to solubilize phosphate, and produce IAA, ACC deaminase and siderophores [49,50,51].

Meanwhile, the indirect mechanism is mainly due to their biocontrol activities attributed to the production of antimicrobial compounds in response to biotic stress [52]. The members of OG*Ba* produced antimicrobial compounds such as hydrogen cyanide (HCN) and cyclic lipopeptides such as surfactin used to inhibit the growth of pathogenic microbes [53,54]. The interactions of biocontrol agents with plant roots enhance plant resistance against some competing microbes including pathogenic bacteria, fungi and viruses. This phenomenon is termed as induced systemic resistance (ISR) [6,55].

The members of OG*Ba* were proven to provide advantages to the agricultural sector by contributing to plant pathogen disease suppression. In plant disease management, the members of OG*Ba* acted as plant growth-promoting bacteria (PGPB) that aid in the development of plants and reduce the proliferation of plant pathogens (Table 2). The secretion of antimicrobial compounds such as surfactin from PGPB was suggested to trigger the pathways of ISR which contributed to the suppressive effect of plant immunity [56,57]. Surfactin was determined to act as elicitors of plant immunity and enhance resistance towards further pathogenesis in plants [47]. In the lettuce rhizosphere, increased production of surfactin by *B. velezensis* FZB42 in the axenic system was suggested to contribute to the disease suppression towards *Rhizoctonia solani* infection [53]. Similarly, the treatment using *B. velezensis* FZB42 in tobacco plants was suggested improve ISR and enhance plant height and fresh weight, while lowering the disease severity rating of the tobacco mosaic virus (TMV) [58].

Bacterial species from the OG*Ba* are used in bioformulations. For instance, the bacterial strain *B. velezensis* FZB42 had been established as a model strain for plant growth promotion and as a biocontrol agent [55]. In 2019, tomato seeds coated with gum arabic as adhesive along with liquid bioformulations containing *B. velezensis* FZB42 showed great inhibitory effects against *Fusarium solani* infections under in vitro conditions. Increments in germination percentage and germination rate as compared with the control were also reported [81].

To date, there are a few bioformulations containing bacterial species from the OG*Ba* available on the market (Table 3), such as SERENADE^®^ (Bayer Crop Science, Germany) which contains *B. velezensis* QST 713 (previously *B. subtilis* QST 713) and Double Nickel 55^TM^ (Certis Columbia, MD USA) which contains *B. velezensis* D747 (previously *B. amyloliquefaciens* D747) [55]. The application of SERENADE^®^ together with Fracture fungicide (CEV, Portugal), which contains BLAD polypeptide, had shown notable success in controlling *Botrytis* blossom blight disease infection in blueberries [82]. Application of Double Nickel 55^TM^ was found to be effective in controlling white mold in snap beans caused by *Sclerotinia sclerotiorum*. Double Nickel 55^TM^, a biofungicide, was approved for organic vegetable production by the National Organic Program and Organic Materials Review Institute [83].

Apart from the aforementioned uses, the members of OG*Ba* have also been applied as biocontrol agents against parasitic nematodes and protist pathogens. In 2008, *B. velezensis* FZB42 was reported to reduce nematode eggs in roots, juvenile worms in soil and plant galls on tomato [84]. Genomic study revealed that the whole genome of *B. velezensis* FZB42 encoded a diverse spectrum of different antimicrobial compounds able to suppress harmful nematodes living within the plant rhizosphere [85]. In controlling the protist pathogen, *B. velezensis* HB-26 (previously *B. amyloliquefaciens* HB-26) showed promising capability for controlling *Plasmodiophora brassicae*, a root-infecting protist that causes clubroot disease in brassica species. Many antimicrobial compounds showing specific activities against *P. brassicae* were found in the genome of *B. velezensis* HB-26 [86]. Overall, much more focus is still needed to fulfill the understanding of the molecular basis for the ability of members of OG*Ba* to inhibit nematodes and protists beyond in silico genomic studies. Understanding such attributes will help to shed light on the functionalities as well as the biological roles of antimicrobial compounds from OG*Ba* not only for improved plant growth but as biocontrol agents to minimize the proliferation of plant pathogens including viruses, bacteria, fungus, nematodes, and protists.

### 3.2. Source of Commercial Enzymes

Microbial enzymes such as *α*-amylase, protease, and lipase have been used in various biotechnological applications including textile applications, feed industry, food industry, and organic synthesis [87,88,89]. The U.S. Food and Drug Administration (FDA) in 1999 reported that enzymes such as *α*-amylase and protease originating from *B. subtilis* are Generally Recognized as Safe (GRAS) for use as direct food ingredients [90]. As members of the *B. subtilis* species complex, OG*Ba* bacteria are a potent bacterial group due to their abilities to produce various types of enzymes including *α*-amylase, protease, lipase, cellulase, xylanase, pectinase, aminotransferase, barnase, peroxidase, and laccase (Table 4).

### 3.3. Antimicrobial Compounds Producer

**Figure 3:**
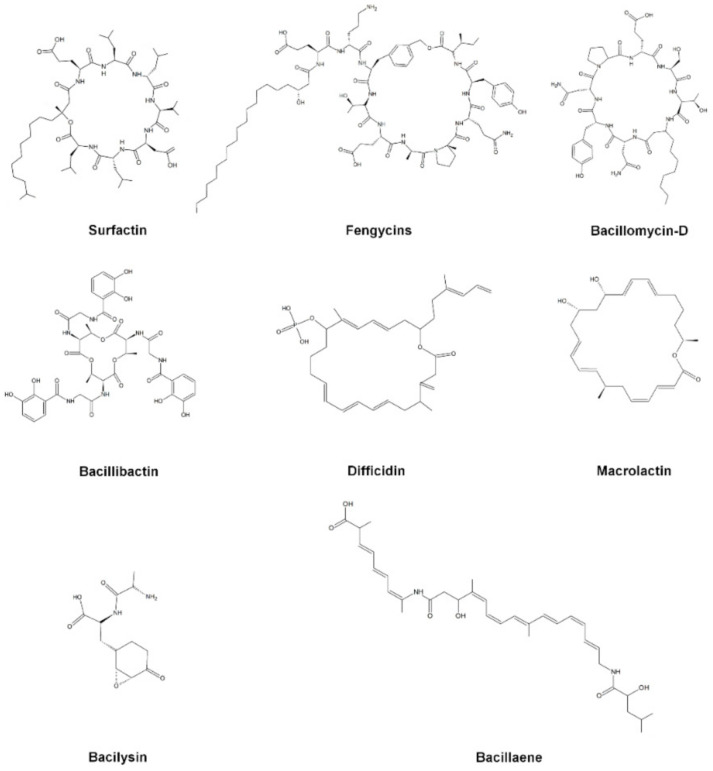
Antimicrobial compounds produced by members of the operational group *Bacillus amyloliquefaciens.*

The increment in the global antibiotic-resistant pathogens has led to the exploration of compounds with alternative therapeutic strategies [104]. The members of OG*Ba* were reported to produce antimicrobial compounds used in the suppression of pathogens [45]. The antimicrobial compounds produced by the member of OG*Ba* have been reviewed previously [8,105]. The members of OG*Ba* produced some important antimicrobial compounds (Figure 3), including non-ribosomal peptides (surfactin, fengycin, bacillomycin-D, bacilysin and bacillibactin) and polyketides (bacillaene, macrolactin and difficidin) [6,105].

Non-ribosomal peptides produced by bacteria and fungi contain two or more moieties derived from amino acids [106]. The mode of action of non-ribosomal peptides involves the disruption to the cell membrane and inhibition on the transfer of peptidoglycan precursors to bactoprenol pyrophosphate [107]. In 2019, surfactins from *B. velezensis* 9D-6 were found to inhibit the in vitro growth of bacteria (*B. cereus*, *C. michiganensis*, *Pantoea agglomerans*, *Ralstonia solanacearum*, *Xanthomonas campestris* and *Xanthomonas euvesicatoria*) and fungi (*Alternaria solani*, *Cochliobolus carbonum*, *F. oxysporum*, *F. solani*, *Gibberella pulicaris*, *Gibberella zeae*, *Monilinia fructicola*, *Pyrenochaeta terrestris* and *R. solani*) pathogens [108]. In another related study*,* in silico genomic study of *B. siamensis* JFL15 had gene clusters involved in the biosynthesis of antimicrobial compounds. The LC–MS/MS analysis confirmed the presence of iturin A and bacillomycin F. Both compounds showed strong antifungal activities against *Magnapothe grisea*, *R. solani* and *Colletotrichum gloeosporioides*, as analyzed under in vitro conditions [109]. Moreover, the presence of fengycin, bacilysin, and bacillibactin had also been reported from *B. velezensis* OSY-S3 that showed inhibition activities against *Listeria innocua*, *Escherichia coli*, *Penicillium* sp., *Cladosporium* sp., and *Staphylococcus aureus* [110].

Polyketides are biopolymers of acetate and other short carboxylates that are biosynthesized by polyketide synthases, a natural metabolite produced by microorganisms and plants which possess various antifungal and antibacterial activities [111,112]. Since the discovery of polyketides (e.g., streptomycin in 1950), the exploration of new polyketides has assisted pharmaceutical companies in isolating new antibiotic-producing strains as the main sources of antibiotics [113]. Antibacterial polyketides including bacillaene, macrolactin and difficidin were reported from *B. velezensis* OSY-GA1 [109]. Moreover, *B. velezensis* YJ11-1-4 isolated from doenjang exhibited good antimicrobial activities against bacterial (*B. cereus*, *E. coli*, *Listeria monocytogenes* and *S. aureus*) and fungal (*Aspergillus flavus* subsp. *flavus*) foodborne pathogens. Genomic analysis reveals the presence of antibiotic biosynthesis operons including bacillaene, macrolactin and difficidin in the genome of *B. velezensis* OSY-GA1 [114]. Additionally, four new glycosylated macrolactin compounds, namely macrolactins O, P, Q and R, had been isolated from the liquid cultures of *B. velezensis* AH159-1. These compounds inhibited *S. aureus* peptide deformylase and also showed antibacterial activities against *E. coli* and *S. aureus* [115].

### 3.4. Potential as Probiotics

Probiotics are live microbial feed supplements that benefit the host animal by improving the microbial balance. Probiotics have become increasingly popular due to continuously expanding scientific evidence pointing to their beneficial effects on both humans and animals [116]. Within the OG*Ba*, some *B. velezensis* strains are reported to display probiotic potential and have been applied as probiotics for animals [117]. For instance, *B. velezensis* H57 (previously *B. amyloliquefaciens* H57) isolated from lucerne was first investigated in the research to prevent fungal spoilage of hay [118]. Because it is an endospore-forming bacterium able to produce antimicrobial compounds, *B. velezensis* H57 was commercialized as a spoilage control agent under the product name HayRite™ (Biocare and BASF, Australia). Interestingly, sheep and cattle fed on HayRite™ showed improvements in digestibility and nitrogen retention leading to increased weight gain [118]. Genomically, the potential of *B. velezensis* H57 to synthesize antimicrobial compounds including surfactin (*srfABCD*), iturin (*ituABCD*), bacillomycin D (*bmyABC*), fengycin (*fenABCDE*), macrolactin (*mlnABCDEFGHI*), difficidin (*dfnABCDEFGHIJ*) and bacillaene (*baeEDLMNJRS*) were suggested to facilitate the probiotic effects of *B. velezensis* H57 [27]. In another related study, *B. velezensis* FTC01 manifested itself as a probiotic [119]. Genes coding for hydrolases (peptidases, phytases and glycosidases) that can improve feed digestion and prevent intestinal disorders are present in the genome of *B. velezensis* FTC01. Additionally, peptidylprolyl isomerase (*prsA*) gene (a gene that is involved in bacterial adhesion and signaling of biofilm formation in the host gut) was also found. Moreover, in silico genome analysis of *B. velezensis* FTC01 proved the presence of gene clusters involved in the synthesis of antimicrobial peptides. Similarly, gene clusters involved in the synthesis of antimicrobial peptides were also found in the genome of *B. velezensis* JT3-1, a probiotic strain isolated from faeces of the domestic yak [21]. The antimicrobial activity of *B. velezensis* JT3-1 was confirmed using an antimicrobial assay. Strain JT3-1 manifested strong antagonistic activities against various intestinal pathogenic flora including *L. monocytogenes*, *S. aureus*, *E. coli*, *Salmonella typhimurium*, *Mannheimia haemolytica*, *Staphylococcus hominis*, *Clostridium perfringens* and *Mycoplasma bovis*.

*B. velezensis* B-1895 (previously *B. amyloliquefaciens* B-1895) has been commercially used as a probiotic in the fish industry, particularly for *Alburnus leobergi* [120,121]. Its probiotic potential was proven thought the Ames test (reported as non-mutagenic) and antimicrobial activities (against *Streptococcus intermedius* and *Porphyromonas gingivalis*). Moreover, the endospores of *B. velezensis* B-1895 were found tolerant to 0.3% (*w/v*) bile salts and survived incubation for 4 h in MRS broth at pH 2.0–3.0. Overall, the results suggested the potential of *B. velezensis* B-1895 as a fish probiotic [122]. In another related study, *B. velezensis* JW also manifested itself as a fish probiotic [123]. Strain JW showed antibacterial activities against a broad range of bacterial fish pathogens (*Aeromonas hydrophila*, *Aeromonas salmonicida*, *Lactococcus garvieae*, *Streptococcus agalactiae* and *Vibrio parahemolyticus*). Dietary administration of *B. velezensis* JW induced an immune response in *Carassius auratus*. The immune-related genes in *C. auratus* such as interferon gamma gene (IFN- *γ*), tumor necrosis factor-*α* (TNF-*α*), interleukin-1 (IL-1), interleukin-4 (IL-4) and interleukin-10 (IL-10) were found to be upregulated by *B. velezensis* JW-supplemented diets. It is noteworthy that *C. auratus* fed with *B. velezensis* JW-supplemented diets showed improvements in survival rate after *A. hydrophila* infection. This was supported genomically by the presence of antimicrobial gene clusters in the genome of *B. velezensis* JW [122]. Moreover, a potential probiotic effect of *B. velezensis* V4 on the growth performance of *Oncorhynchus mykiss* had also been investigated [124]. Cell-free supernatant of *B. velezensis* V4 with anti-*A. salmonicida* was shown to contain antimicrobial compounds including iturin, macrolactin and difficidin. The mortality rate of *O. mykiss* was reduced by 27% and the weight gain ratio was increased by 71% through the 1% (*v/w*) addition of *B. velezensis* V4. Overall, the findings demonstrated that *B. velezensis* V4 was an effective probiotic in *O. mykiss*.

The commercialization of *B. amyloliquefaciens* as a probiotic in aquaculture is not as common compared to its agricultural applications (Table 3). Ecobiol^®^ Soluble Plus, is one of the commercial probiotic products reported as containing *B. amyloliquefaciens* at a concentration of 10^9^ CFU/g, specifically formulated for applications in poultry and swine, as well as in aquaculture. There was research conducted on the commercial probiotic Ecobiol^®^ Soluble to observe its positive effects on the biofloc culture of *Litopenaeus vannamei* and its benefits on water quality, growth performance and the immune system of shrimps. Three doses of probiotic (9.48 × 10^4^, 1.90 × 10^5^ and 3.79 × 10^5^ CFU/g) were applied to the culture water for 42 days. At the end of the trial, there was no significant improvement in the water quality. However, it showed notable changes in the immune system of the shrimp. As compared to the control treatment, there was an increase in the total protein concentration and granular hemocytes, and a decrease in the cell number with apoptosis in the hemolymph in all treatments. Therefore, other than being mixed with feed, *B. amyloliquefaciens* in the commercial probiotic Ecobiol^®^ Soluble Plus could also be applied directly to the culture system; this research proved it provided better resistance to shrimps against the outbreak of pathogens in shrimp biofloc systems [125].

There is much ongoing research on the development and formulations of bacterial strains belonging to the OG*Ba* as potential probiotics for commercialization purposes in the aquaculture industry. Most of the studies have emphasized probiotic feed formulations, feeding trials on a small scale before moving to field trials. For instance, dietary inclusion of *B. amyloliquefaciens* at 10^6^ CFU/g fed to zebra fish improved the expression levels of metabolism-related genes, enzyme activities and oxidative stress-related genes in the fish liver as well as enhanced their immune resistance against pathogenic *A. hydrophila* and *S. agalactiae*. In addition, the strain of *B*. *amyloliquefaciens* used in this study was able to express recombinant xylanase, an important enzyme that aided in better feed digestibility and efficiency [126]. In another related study, the administration of *B. amyloliquefaciens* (1 × 10^9^ CFU/g), together with *Spirulina platensis* in formulated diet for tilapia, improved growth performance and feed utilization after a 60 day feeding trial. The mRNA level of the *TNF-α* gene and the transcription of *SOD* were considerably higher in tilapia fed with dietary *B. amyloliquefaciens* and *S. platensis* compared to the control group [127]. Moreover, *B. amyloliquefaciens* at a concentration of 10^6^ CFU/mL provided significant protection to juvenile blue swimming crabs, *Portunus pelagicus*, when challenged with *Vibrio harveyi* in in vivo trials [128]. Nevertheless, further studies are necessary, mainly on probiotic formulation along with larger field trials, to strengthen the outcomes in order to be able to commercialize bacterial strains belonging to the OG*Ba* for aquaculture use.

In vivo and field trials are critical in probiotic development. Occasionally, there were negative outcomes in in vivo studies which were carried out based upon the positive results acquired from the preliminary in vitro assays, which indicated the possibility of negative correlations between trials in vitro and in vivo. Hence, it is crucial to understand and to optimize various conditions in in vivo studies or field trials including the probiotic formulation which may affect the survival, colonization, proliferation, and interaction of the probiotic with the host in a certain environment [129].

### 3.5. Potential as Bioremediation Agents

The use of microorganisms as bioremediation agents has become a burgeoning trend [130]. To date, most research focused on the plant growth-promoting activity and antimicrobial compounds of OG*Ba* is as described above. Interestingly, in 2019, *B. amyloliquefaciens* YP6 was reported to exhibit both plant growth-promoting activity and broad-spectrum organophosphorus pesticide (OP) removal [131]. In silico genome analysis of *B. amyloliquefaciens* YP6 found it to contain a variety of promising genes, including phosphorus solubilizing and OP-degrading related genes (*pho*D, *pho*A, *phr*C, *pho*E, *ycs*E, *bcr*C and *yva*K), indole-3-acetic acid synthesis related genes (*amh*X, *cge*E and *eps*M), and siderophores synthesis related genes (*ent*B, *men*F, *ent*C and *ent*A). The results hinted at the potential application of *B. amyloliquefaciens* YP6 in agricultural and environmental remediations. Overall, much more focus is still needed to understand the OP-degrading related genes beyond in silico genome analysis. Therefore, it is necessary to conduct further studies to determine the in vitro functional genomics and the OP-degrading enzyme activities of the members of OG*Ba*. Understanding such attributes will help to shed light on the applicability of the OG*Ba* in OPs degradation and in the bioremediation processes as a whole.

## 4. Concluding Remark and Future Perspectives

In conclusion, the progress of the research on the biotechnological applications of bacterial species that belong to OG*Ba* is remarkable. The bacteria are important not only industrially, but also environmentally. A plethora of studies have addressed the abilities of the members of OG*Ba* as plant growth-promoters, biocontrol agents, probiotics, bioremediation agents as well as producers of commercial enzymes and antibiotics. Moreover, the use of the bacteria in optimized bioformulations as well as the demonstration of the great success of the commercialized products give us hope towards more sustainable agricultural and aquacultural industries. Owing to the listed biotechnological applications and potentials, more research should be done focusing on the integration of system biology data derived from genomics, phenomics, proteomics, metabolomics and fluxomic analyses in order to expand our basic understanding on the versatility of the members of OG*Ba*. Enabling the prediction of cellular functions and metabolites produced by the members of this operational group could provide fundamental knowledge towards the enhancement of the applications of their potentials in biotechnology and bioprocessing for the benefit of all.

## Figures and Tables

**Table 1:** Characterizations of bacterial species under the operational group *Bacillus amyloliquefaciens.*

Characterization		*B. amyloliquefaciens*	*B. siamensis*	*B. velezensis*	*B. nakamurai*
Type Strain		DSM 7^T^ / ATCC 23350^T^ / F^T^	KCTC 13613^T^ / PD-A10^T^ / BCC 22614^T^	NRRL B-23189^T^ / CR-502^T^ / CECT 5686^T^ / LMG 22478^T^	NRRL B-41091^T^ / CCUG 68786^T^
Isolation Source		Soil and industrial *α*-amylase fermentations	Salted crab (*poo-khem*) in Thailand	Brackish water sample from the river Velez at Torredelmar in Ma’laga, southern Spain	Soil in Tierra del Fuego, Argentina
Size		0.7–0.9 × 1.8–3.0 µm	0.3–0.6 × 1.5–3.5 µm	0.5 × 1.5–3.5 µm	0.74–0.93 × 1.39–2.04 µm
Endospore		Oval spores are central or paracentral in unswollen sporangia	Ellipsoidal spores are central or sub-terminal positions in swollen sporangia	Ellipsoidal spores are paracentral or sub-terminal positions in unswollen sporangia	Ellipsoidal spores are central in unswollen sporangia
G + C Content (mol %)		44.6	41.4	46.1–46.4	43.8
Growth Temperature		Optimal growth temperature is 30–40 °C. No growth occurs below 15 °C or above 50 °C.	Optimal growth temperature is 37 °C. Growth occurs at 4 °C and 55 °C.	Grow within the temperature range of 15–45 °C	Grow within the temperature range of 17–50 °C, with an optimum of 37 °C
NaCl Resistance (*w/v*)		Growth occurs with 0–10% NaCl	Growth occurs with 0–14% NaCl	Growth occurs with 0–12% NaCl	Growth occurs with 0–9% NaCl
Substrate Utilization	Tyrosine	-	-	-	+
	Citrate	+	-	-	+
Fermentation (acid)	Lactose	+	+	+	-
	Trehalose	+	-	+	+
Reference		[1]	[16]	[17]	[18]

Note: All the bacterial species are able to metabolize casein, gelatin, starch, fructose, cellobiose, glucose, glycerol, maltose, mannitol, raffinose, salicin and sucrose. Symbol: +, positive result; -, negative result.

**Table 2:** Plant pathogen suppression by members of the operational group *Bacillus amyloliquefaciens* in various plant species.

PGPB Strain	Disease and Pathogen	Plant Species	Reference
*B. siamensis* KCTC 13613	*R. solani* *Botrytis cinerea* *Micrococcus luteus*	*Arabidopsis thaliana*	[59]
*B. velezensis* 83	Anthracnose disease	*Zea mays* *A. thaliana*	[20]
*B. velezensis* 1B-23	*Clavibacter michiganensis* subsp. *michiganensis*	*Solanum lycopersicum*	[60]
*B. velezensis* B25	*Fusarium verticillioides*	*Z. mays*	[61]
*B. velezensis* BTLK6A	*Magnaporthe oryzae Triticum*	*Triticum aestivum*	[62]
*B. velezensis* BTS 4			
*B. velezensis* CC09	Powdery mildew disease	*T. aestivum*	[28]
*B. velezensis* CGMCC 11640	*Botryosphaeria dothidea*	*Carya cathayensis*	[63]
*B. velezensis* Co1-6	*Verticillium dahliae* *R. solani* *Fusarium culmorum* *Ralstonia solanacearum*	*Matricaria chamomilla*	[64]
*B. velezensis* GB1	*Valsa mali*	*Malus domestica*	[65]
*B. velezensis* GH1-13	*Fusarium fujikuroi* *R. solani* *Xanthmonas oryzae*	*Oryza sativa*	[49]
*B. velezensis* GQJK49	*F. solani*	*Lycium barbarum* L.	[66]
*B. velezensis* GYL4	Anthracnose disease	*Cucumis sativus* L. cv. *Chunsim*	[67]
*B. velezensis* J-5	*B. cinerea*	*S. lycopersicum*	[68]
*B. velezensis* JK	*M. oryzae*	*O. sativa*	[69]
*B. velezensis* L-1	*Botryosphaeria berengeriana*	*Pyrus communis*	[70]
*B. velezensis* LM2303	*Fusarium graminearum*	*T. aestivum*	[71]
*B. velezensis* M27	*Sclerotinia sclerotiorum*	*Lactuca sativa* L.	[72]
*B. velezensis* NJAU-Z9	*Fusarium oxysporum* f. sp. *niveum* *Ralstonia solanacearum*	*Capsicum annuum* L.	[73]
*B. velezensis* NJN-6	*F. oxysporum* f. sp. *cubense*	*Musa* sp.	[74]
*B. velezensis* OEE1	*F. solani*	*Olea europaea* L.	[75]
*B. velezensis* P42	Bacterial wilt and early blight diseases	*S. lycopersicum*	[76]
*B. velezensis* PG12	Apple ring rot disease	*Malus domestica*	[24]
*B. velezensis* TrigoCor1448	*Fusarium* head blight disease	*T. aestivum*	[77]
*B. velezensis* UCMB5113	*Alternaria brassicae* *B. cinerea* *Leptosphaeria maculans* *Verticillium longisporum*	*Brassica napus*	[78]
*B. velezensis* XK-4-1	*Verticillium* wilt disease	*Gossypium* sp.	[79]
*B. velezensis* ZF2	*Corynespora* leaf spot diseases	*C. sativus*	[80]

**Table 3:** Some commercial products containing the members of the operational group *Bacillus amyloliquefaciens* available on the market.

Bacterial Strain	Commercial Product	Company	Description
*B. velezensis* QST 713 (previously *B. subtilis* QST 713)	SERENADE Max	Bayer Crop Science, previously AgraQuest	EPA-registered biofungicide. Controls and suppresses fungal pathogens on foliage and in the soil
	SERENADE SOIL^®^	Bayer Crop Science, previously AgraQuest	EPA-registered biofungicide for food crops
	CEASE^®^	BioWorks, Inc., Victor, New York, U.S.A.	Aqueous suspension biofungicide for leafy and fruiting vegetables, herbs and spices, and ornamentals
*B. velezensis* FZB42 (previously *B. amyloliquefaciens* FZB42)	RhizoVital^®^ 42	ABiTEP GmbH, Berlin, Germany	Biofertilizer, plant-growth-promoting activity, provides protection against various soil-borne diseases
	FZB24^®^ TB	ABiTEP GmbH, Berlin, Ger-many	Plant growth-promoting agent for plant strengthening
	Taegro^®^	Syngenta, Basel, previously Novozyme, Davis, California, and Earth Biosciences	EPA-registered biofungicide for use in North America
*B. velezensis* GB03 (previously *B. subtilis* GB03)	Kodiak™	Bayer Crop Science, North Carolina, NC	EPA-registered biological seed treatment fungicide with demonstrable PGR activity. Efficient in cotton, beans, and vegetables
	Companion	Growth Products Ltd., White Plains, NY	EPA-registered biofungicide that prevents and controls plant diseases
*B. velezensis* D747 (previously *B. amyloliquefaciens* D747)	Double Nickel 55™	Certis Columbia, MD, U.S.A.	EPA-registered biofungicide for control or suppression of fungal and bacterial plant
	Amylo-X^®^	Certis Columbia, MD USA/Intrachem Bio Italia SpA	Biocontrol of *Botrytis* and other fungal diseases of grapes, strawberries, and vegetables, and bacterial diseases, such as fire blight in pome fruit and PSA in kiwi fruit

**Table 4:** Various types of enzymes produced by members of the operational group *Bacillus amyloliquefaciens*.

Bacterial Species	Enzymes	Reference
*B. amyloliquefaciens* KCP2	*α*-amylase and protease	[91]
*B. amyloliquefaciens* NRRL 942	*α*-amylase	[92]
*B. siamensis* JJC33M	*α*-amylase	[93]
*B. velezensis* 157	*α*-amylase, cellulase, xylanase and pectinase	[94]
*B. velezensis* 275	Cellulase, xylanase, peroxidase, and laccase	[95]
*B. velezensis* AP194	Pectinase	[96]
*B. velezensis* AP214	Pectinase	[96]
*B. velezensis* GZB	Laccase	[97]
*B. velezensis* JJ-D34	*α*-amylase, protease and cellulase	[98]
*B. velezensis* Jxnuwx-1	Protease	[99]
*B. velezensis* SB1216	Barnase	[100]
*B. velezensis* SPZ1	Lipase	[101]
*B. velezensis* SYBC H47	Aminotransferase	[102]
*B. velezensis* ZL918	*α*-amylase	[103]

## Data Availability

Not applicable.

## Supplementary Materials

The following are available online at https://www.mdpi.com/2076-2607/9/3/614/s1: Table S1. Bacterial strains from the operational group *Bacillus amyloliquefaciens*.

## References

[1] Priest F.G., Goodfellow M., Shute L.A., Berkeley R.C.W. (1987). *Bacillus amyloliquefaciens* sp. nov., nom. rev. Int. J. Syst. Evol. Microbiol..

[2] Fukumoto J. (1943). Studies on the production of bacterial amylase. I. Isolation of bacteria secreting potent amylases and their distribution. Nippon. Nogeikagaku Kaishi.

[3] Berkeley R.C.W., Logan N.A., Shute L.A., Capey A.G. (1984). Identification of *Bacillus* species. Methods in Microbiology.

[4] Fan B., Blom J., Klenk H.P., Borriss R. (2017). *Bacillus amyloliquefaciens*, *Bacillus velezensis*, and *Bacillus siamensis* form an “operational group *B. amyloliquefaciens*” within the *B. subtilis* species complex. Front. Microbiol..

[5] Dunlap C.A., Kim S.J., Kwon S.W., Rooney A.P. (2016). *Bacillus velezensis* is not a later heterotypic synonym of *Bacillus amyloliquefaciens*; *Bacillus methylotrophicus*, *Bacillus amyloliquefaciens* subsp. plantarum and ‘Bacillus oryzicola’are later heterotypic synonyms of Bacillus velezensis based on phylogenomic. Int. J. Syst. Evol. Microbiol..

[6] Dunlap C.A., Bowman M.J., Rooney A.P. (2019). Iturinic lipopeptide diversity in the *Bacillus subtilis* species group—Important antifungals for plant disease biocontrol applications. Front. Microbiol..

[7] Ye M., Tang X., Yang R., Zhang H., Li F., Tao F., Li F., Wang Z. (2018). Characteristics and application of a novel species of *Bacillus*: *Bacillus velezensis*. ACS Chem. Biol..

[8] Rabbee M.F., Sarafat Ali M., Choi J., Hwang B.S., Jeong S.C., Baek K.H. (2019). *Bacillus velezensis*: A valuable member of bioactive molecules within plant microbiomes. Molecules.

[9] Auch A.F., von Jan M., Klenk H.P., Göker M. (2010). Digital DNA-DNA hybridization for microbial species delineation by means of genome-to-genome sequence comparison. Stand. Genomic Sci..

[10] Connor N., Sikorski J., Rooney A.P., Kopac S., Koeppel A.F., Burger A., Cole S.G., Perry E.B., Krizanc D., Field N.C. (2010). Ecology of speciation in the genus *Bacillus*. Appl. Environ. Microbiol..

[11] Borriss R., Chen X.H., Rueckert C., Blom J., Becker A., Baumgarth B., Fan B., Pukall R., Schumann P., Spröer C. (2011). Relationship of *Bacillus amyloliquefaciens* clades associated with strains DSM7^T^ and FZB42^T^: A proposal for *Bacillus amyloliquefaciens* subsp. amyloliquefaciens subsp. nov. and Bacillus based on complete genome sequence comparisons. Int. J. Syst. Evol. Microbiol..

[12] Chun J., Bae K.S. (2000). Phylogenetic analysis of *Bacillus subtilis* and related taxa based on partial gyrA gene sequences. Antonie Van Leeuwenhoek.

[13] Reva O.N., Dixelius C., Meijer J., Priest F.G. (2004). Taxonomic characterization and plant colonizing abilities of some bacteria related to *Bacillus amyloliquefaciens* and *Bacillus subtilis*. FEMS Microbiol. Ecol..

[14] Ngalimat M.S., Sabri S. (2020). Taxonomic note: Speciation within the operational group *Bacillus amyloliquefaciens* based on comparative phylogenies of housekeeping genes. Asia-Pac. J. Mol. Biol. Biotechnol..

[15] Kumar S., Stecher G., Li M., Knyaz C., Tamura K. (2018). MEGA X: Molecular evolutionary genetics analysis across computing platforms. Mol. Biol. Evol..

[16] Sumpavapol P., Tongyonk L., Tanasupawat S., Chokesajjawatee N., Luxananil P., Visessanguan W. (2010). *Bacillus siamensis* sp. nov., isolated from salted crab (poo-khem) in Thailand. Int. J. Syst. Evol. Microbiol..

[17] Ruiz-García C., Béjar V., Martinez-Checa F., Quesada E. (2005). *Bacillus velezensis* sp nov., a surfactant-producing bacterium isolated from the river velez in malaga, southern Spain. Int. J. Syst. Evol. Microbiol..

[18] Dunlap C.A., Saunders L.P., Schisler D.A., Leathers T.D., Naeem N., Cohan F.M., Rooney A.P. (2016). *Bacillus nakamurai* sp. nov., a black-pigment-producing strain. Int. J. Syst. Evol. Microbiol..

[19] Liu B., Ge B., Azhar N., Zhao W., Cui H., Zhang K. (2018). Complete genome sequence of *Bacillus methylotrophicus* strain NKG-1, isolated from the changbai mountains, China. Genome Announc..

[20] Balderas-Ruíz K.A., Bustos P., Santamaria R.I., González V., Cristiano-Fajardo S.A., Barrera-Ortíz S., Mezo-Villalobos M., Aranda-Ocampo S., Guevara-García Á.A., Galindo E. (2020). *Bacillus velezensis* 83 a bacterial strain from mango phyllosphere, useful for biological control and plant growth promotion. AMB Express.

[21] Li Y., Li X., Jia D., Liu J., Wang J., Liu A., Liu Z., Guan G., Liu G., Luo J. (2019). Complete genome sequence of *Bacillus velezensis* JT3-1, a microbial germicide isolated from yak feces. bioRxiv.

[22] Nannan C., Gillis A., Caulier S., Mahillon J. (2018). Complete genome sequence of *Bacillus velezensis* CN026 exhibiting antagonistic activity against Gram-negative foodborne pathogens. Genome Announc..

[23] Zhang Y., Wang Y., Qin Y., Li P. (2020). Complete genome sequence of *Bacillus velezensis* LPL-K103, an antifungal cyclic lipopeptide bacillomycin L producer from the surface of lemon. 3 Biotech.

[24] Zeng Q., Xie J., Li Y., Chen X., Wang Q. (2019). Draft genome sequence of an endophytic biocontrol bacterium, *Bacillus velezensis* PG12, isolated from apple fruit. Microbiol. Resour. Announc..

[25] Gamez R.M., Rodríguez F., Bernal J.F., Agarwala R., Landsman D., Mariño-Ramírez L. (2015). Genome sequence of the banana plant growth-promoting rhizobacterium *Bacillus amyloliquefaciens* BS006. Genome Announc..

[26] Chen L., Shi H., Heng J., Wang D., Bian K. (2019). Antimicrobial, plant growth-promoting and genomic properties of the peanut endophyte *Bacillus velezensis* LDO2. Microbiol. Res..

[27] Schofield B.J., Skarshewski A., Lachner N., Ouwerkerk D., Klieve A.V., Dart P., Hugenholtz P. (2016). Near complete genome sequence of the animal feed probiotic, *Bacillus amyloliquefaciens* H57. Stand. Genomic Sci..

[28] Cai X., Kang X., Xi H., Liu C., Xue Y. (2016). Complete genome sequence of the endophytic biocontrol strain *Bacillus velezensis* CC09. Genome Announc..

[29] Geng W., Cao M., Song C., Xie H., Liu L., Yang C., Feng J., Zhang W., Jin Y., Du Y. (2011). Complete genome sequence of *Bacillus amyloliquefaciens* LL3, which exhibits glutamic acid-independent production of poly-γ-glutamic acid. J. Bacteriol..

[30] Deng Q., Wang R., Sun D., Sun L., Wang Y., Pu Y., Fang Z., Xu D., Liu Y., Ye R. (2020). Complete genome of *Bacillus velezensis* CMT-6 and comparative genome analysis reveals lipopeptide diversity. Biochem. Genet..

[31] Khalaf E.M., Raizada M.N. (2020). Draft genome sequences of *Bacillus* and *Paenibacillus* species isolated from seeds of *Citrullus lanata* (watermelon), *Cucurbita moschata* (butternut squash), and *Cucurbita pepo* L. var pepo L. (pumpkin). Microbiol. Resour. Announc..

[32] Zhao X., Zhou Z., Han Y., Wang Z., Fan J., Xiao H. (2013). Isolation and identification of antifungal peptides from *Bacillus* BH072, a novel bacterium isolated from honey. Microbiol. Res..

[33] Ngalimat M.S., Rahman R.N.Z.R.A., Yusof M.T., Syahir A., Sabri S. (2019). Characterisation of bacteria isolated from the stingless bee, *Heterotrigona itama*, honey, bee bread and propolis. PeerJ.

[34] Zulkhairi Amin F.A., Sabri S., Ismail M., Chan K.W., Ismail N., Mohd Esa N., Mohd Lila M.A., Zawawi N. (2020). Probiotic properties of *Bacillus* strains isolated from stingless bee (*Heterotrigona itama*) honey collected across Malaysia. Int. J. Environ. Res. Public Health.

[35] Kalinowski J., Ahrens B., Al-Dilaimi A., Winkler A., Wibberg D., Schleenbecker U., Rückert C., Wölfel R., Grass G. (2018). Isolation and whole genome analysis of endospore-forming bacteria from heroin. Forensic Sci. Int. Genet..

[36] Lim S.B.Y., Junqueira A.C.M., Uchida A., Purbojati R.W., Houghton J.N.I., Chénard C., Wong A., Kolundžija S., Clare M.E., Kushwaha K.K. (2018). Genome sequence of *Bacillus velezensis* SGAir0473, isolated from tropical air collected in Singapore. Genome Announc..

[37] Guo W., Cui P., Chen X. (2015). Complete genome of *Bacillus* sp. Pc3 isolated from the Antarctic seawater with antimicrobial activity. Mar. Genomics.

[38] Agersø Y., Stuer-Lauridsen B., Bjerre K., Jensen M.G., Johansen E., Bennedsen M., Brockmann E., Nielsen B. (2018). Antimicrobial susceptibility testing and tentative epidemiological cutoff values for five *Bacillus* species relevant for use as animal feed additives or for plant protection. Appl. Enviromental Microbiol..

[39] Pan H.Q., Li Q.L., Hu J.C. (2017). The complete genome sequence of *Bacillus velezensis* 9912D reveals its biocontrol mechanism as a novel commercial biological fungicide agent. J. Biotechnol..

[40] Guo Y., Zhou J., Tang Y., Ma Q., Zhang J., Ji C., Zhao L. (2020). Characterization and genome analysis of a zearalenone-degrading *Bacillus velezensis* strain ANSB01E. Curr. Microbiol..

[41] Wu J., Xu G., Jin Y., Sun C., Zhou L., Lin G., Xu R., Wei L., Fei H., Wang D. (2018). Isolation and characterization of *Bacillus* sp. GFP-2, a novel *Bacillus* strain with antimicrobial activities, from whitespotted bamboo shark intestine. AMB Express.

[42] Molinatto G., Franzil L., Steels S., Puopolo G., Pertot I., Ongena M. (2017). Key impact of an uncommon plasmid on *Bacillus amyloliquefaciens* subsp. plantarum S499 developmental traits and lipopeptide production. Front. Microbiol..

[43] Feng J., Gu Y., Wang J., Song C., Yang C., Xie H., Zhang W., Wang S. (2013). Curing the plasmid pMC1 from the poly (γ-glutamic acid) producing *Bacillus amyloliquefaciens* LL3 strain using plasmid incompatibility. Appl. Biochem. Biotechnol..

[44] Chun B.H., Kim K.H., Jeong S.E., Jeon C.O. (2019). Genomic and metabolic features of the *Bacillus amyloliquefaciens* group– *B. amyloliquefaciens*, *B. velezensis*, and *B. siamensis*– revealed by pan-genome analysis. Food Microbiol..

[45] Belbahri L., Chenari Bouket A., Rekik I., Alenezi F.N., Vallat A., Luptakova L., Petrovova E., Oszako T., Cherrad S., Vacher S. (2017). Comparative genomics of *Bacillus amyloliquefaciens* strains reveals a core genome with traits for habitat adaptation and a secondary metabolites rich accessory genome. Front. Microbiol..

[46] Kashyap B.K., Solanki M.K., Pandey A.K., Prabha S., Kumar P., Kumari B. (2019). Bacillus as plant growth promoting rhizobacteria (PGPR): A promising green agriculture technology. Plant Health under Biotic Stress.

[47] Chowdhury S.P., Hartmann A., Gao X., Borriss R. (2015). Biocontrol mechanism by root-associated *Bacillus amyloliquefaciens* FZB42—A Review. Front. Microbiol..

[48] Sibponkrung S., Kondo T., Tanaka K., Tittabutr P., Boonkerd N., Yoshida K.I., Teaumroong N. (2020). Co-inoculation of *Bacillus velezensis* strain S141 and *Bradyrhizobium* strains promotes nodule growth and nitrogen fixation. Microorganisms.

[49] Kim S.Y., Song H., Sang M.K., Weon H.Y., Song J. (2017). The Complete genome sequence of *Bacillus velezensis* strain GH1-13 reveals agriculturally beneficial properties and a unique plasmid. J. Biotechnol..

[50] Lu K., Jin Q., Lin Y., Lu W., Li S., Zhou C., Jin J., Jiang Q., Ling L., Xiao M. (2020). Cell-free fermentation broth of *Bacillus velezensis* strain S3-1 improves pak choi nutritional quality and changes the bacterial community structure of the rhizosphere soil. Front. Microbiol..

[51] Wang C., Zhao D., Qi G., Mao Z., Hu X., Du B., Liu K., Ding Y. (2020). Effects of *Bacillus velezensis* FKM10 for promoting the growth of *Malus hupehensis* rehd. and inhibiting Fusarium verticillioides. Front. Microbiol..

[52] Kumar A., Kumar R., Kumari M., Goldar S. (2020). Enhancement of plant growth by using PGPR for a sustainable agriculture: A review. Int. J. Curr. Microbiol. Appl. Sci..

[53] Chowdhury S.P., Uhl J., Grosch R., Alquéres S., Pittroff S., Dietel K., Schmitt-Kopplin P., Borriss R., Hartmann A. (2015). Cyclic lipopeptides of *Bacillus amyloliquefaciens* subsp. plantarum colonizing the lettuce rhizosphere enhance plant defense responses toward the bottom rot pathogen Rhizoctonia solani. Mol. Plant-Microbe Interact..

[54] Li B., Li Q., Xu Z., Zhang N., Shen Q., Zhang R. (2014). Responses of beneficial *Bacillus amyloliquefaciens* SQR9 to different soilborne fungal pathogens through the alteration of antifungal compounds production. Front. Microbiol..

[55] Fan B., Wang C., Song X., Ding X., Wu L., Wu H., Gao X., Borriss R. (2018). *Bacillus velezensis* FZB42 in 2018: The Gram-positive model strain for plant growth promotion and biocontrol. Front. Microbiol..

[56] Doornbos R.F., van Loon L.C., Bakker P.A. (2012). Impact of root exudates and plant defense signaling on bacterial communities in the rhizosphere. a review. Agron. Sustain. Dev..

[57] Erlacher A., Cardinale M., Grosch R., Grube M., Berg G. (2014). The impact of the pathogen *Rhizoctonia solani* and its beneficial counterpart *Bacillus amyloliquefaciens* on the indigenous lettuce microbiome. Front. Microbiol..

[58] Wang S., Wu H., Qiao J., Ma L., Liu J., Xia Y., Gao X. (2009). Molecular mechanism of plant growth promotion and induced systemic resistance to Tobacco Mosaic Virus by *Bacillus* spp.. J. Microbiol. Biotechnol..

[59] Jeong H., Jeong D.E., Kim S.H., Song G.C., Park S.Y., Ryu C.M., Park S.H., Choi S.K. (2012). Draft genome sequence of the plant growth-promoting bacterium *Bacillus siamensis* KCTC 13613^T^. J. Bacteriol..

[60] Laird M., Piccoli D., Weselowski B., McDowell T., Renaud J., MacDonald J., Yuan Z.C. (2019). Surfactin-producing *Bacillus velezensis* 1B-23 and *Bacillus* sp. 1D-12 protect tomato against bacterial canker caused by *Clavibacter michiganensis* subsp. michiganensis. J. Plant Pathol..

[61] Douriet-Gámez N.R., Maldonado-Mendoza I.E., Ibarra-Laclette E., Blom J., Calderón-Vázquez C.L. (2018). Genomic analysis of Bacillus sp. strain B25, a biocontrol agent of maize pathogen *Fusarium verticillioides*. Curr. Microbiol..

[62] Dutta S., Surovy M.Z., Gupta D.R., Mahmud N.U., Chanclud E., Win J., Kamoun S., Islam T. (2018). Genomic analyses reveal that biocontrol of wheat blast by *Bacillus* spp. may be linked with production of antimicrobial compounds and induced systemic resistance in host plants. Figshare.

[63] Cheng M., Xu Q., Li Y., Qin H., Chen J. (2016). Antifungal activity and identification of active compounds of *Bacillus amyloliquefaciens* subsp. plantarum against Botryosphaeria dothidea. For. Pathol..

[64] Köberl M., White R.A., Erschen S., Spanberger N., El-Arabi T.F., Jansson J.K., Berg G. (2015). Complete genome sequence of *Bacillus amyloliquefaciens* strain Co1-6, a plant growth-promoting rhizobacterium of *Calendula officinalis*. Genome Announc..

[65] Zhang J.X., Gu Y.B., Chi F.M., Ji Z.R., Wu J.Y., Dong Q.L., Zhou Z.S. (2015). *Bacillus amyloliquefaciens* GB1 can effectively control apple valsa canker. Biol. Control.

[66] Ma J., Liu H., Liu K., Wang C., Li Y., Hou Q., Yao L., Cui Y., Zhang T., Wang H. (2017). Complete genome sequence of *Bacillus velezensis* GQJK49, a plant growth- promoting rhizobacterium with antifungal activity. Genome Announc..

[67] Kim J.D., Jeon B.J., Han J.W., Park M.Y., Kang S.A., Kim B.S. (2016). Evaluation of the endophytic nature of *Bacillus amyloliquefaciens* strain GYL4 and its efficacy in the control of anthracnose. Pest Manag. Sci..

[68] Jia Z., Jin W., Huang Y., Song S. (2017). Complete genome sequence of *Bacillus subtilis* J-5, a potential biocontrol agent. Genome Announc..

[69] Jing R., Li N., Wang W., Liu Y. (2020). An endophytic strain JK of genus *Bacillus* isolated from the seeds of super hybrid rice (*Oryza sativa* L., Shenliangyou 5814) has antagonistic activity against rice blast pathogen. Microb. Pathog..

[70] Sun P., Cui J., Jia X., Wang W. (2017). Complete genome sequence of *Bacillus velezensis* L-1, which has antagonistic activity against pear diseases. Genome Announc..

[71] Chen L., Heng J., Qin S., Bian K. (2018). A Comprehensive understanding of the biocontrol potential of *Bacillus velezensis* LM2303 against *Fusarium* head blight. PLoS ONE.

[72] Lee S.Y., Kim B.Y., Ahn J.H., Song J., Seol Y.J., Kim W.G., Weon H.Y. (2012). Draft genome sequence of the biocontrol bacterium *Bacillus amyloliquefaciens* strain M27. J. Bacteriol..

[73] Zhang Y., Gao X., Wang S., Zhu C., Li R., Shen Q. (2018). Application of *Bacillus velezensis* NJAU-Z9 enhanced plant growth associated with efficient rhizospheric colonization monitored by QPCR with primers designed from the whole genome sequence. Curr. Microbiol..

[74] Yuan J., Raza W., Shen Q., Huang Q. (2012). Antifungal activity of *Bacillus amyloliquefaciens* NJN-6 volatile compounds against *Fusarium oxysporum* f. sp. cubense. Appl. Environ. Microbiol..

[75] Cheffi M., Bouket A.C., Alenezi F.N., Luptakova L., Belka M., Vallat A., Rateb M.E., Tounsi S., Triki M.A., Belbahri L. (2019). Olea Europaea L. Root endophyte *Bacillus velezensis* OEE1 counteracts oomycete and fungal harmful pathogens and harbours a large repertoire of secreted and volatile metabolites and beneficial functional genes. Microorganisms.

[76] Rathna V. (2018). Exploiting the Bio Control Potentiality of *Bacillus velezensis* P42 and A6 Strains Against Important Bacterial Wilt and Early Blight Diseases of Tomato. Master’s Thesis.

[77] Nelson B.A., Ramaiya P., de Leon A.L., Kumar R., Crinklaw A., Jolkovsky E., Crane J.M., Bergstrom G.C., Rey M.W. (2014). Complete genome sequence for the *Fusarium* head blight antagonist *Bacillus amyloliquefaciens* strain TrigoCor 1448. Genome Announc..

[78] Niazi A., Manzoor S., Asari S., Bejai S., Meijer J., Bongcam-Rudloff E. (2014). Genome analysis of *Bacillus amyloliquefaciens* subsp. plantarum UCMB5113: A rhizobacterium that improves plant growth and stress management. PLoS ONE.

[79] Sun Z., Hsiang T., Zhou Y., Zhou J. (2015). Draft genome sequence of *Bacillus amyloliquefaciens* XK-4-1, a plant growth-promoting endophyte with antifungal activity. Genome Announc..

[80] Xu S., Xie X., Zhao Y., Shi Y., Chai A., Li L., Li B. (2020). Whole-genome analysis of *Bacillus velezensis* ZF2, a biocontrol agent that protects *Cucumis sativus* against corynespora leaf spot diseases. 3 Biotech.

[81] David Paul Raj R.S., Beena Kanimozhi R., Gomez L.A., Rohini S. (2019). Evaluation of biocontrol efficacy of herbal and bioformulations against root rot pathogen *Fusarium solani* in tomato. Int. J. Recent Technol. Eng..

[82] De Curtis F., Ianiri G., Raiola A., Ritieni A., Succi M., Tremonte P., Castoria R. (2019). Integration of biological and chemical control of brown rot of stone fruits to reduce disease incidence on fruits and minimize fungicide residues in juice. Crop Prot..

[83] Pethybridge S.J., Gugino B.K., Kikkert J.R. (2019). Efficacy of Double Nickel LC (*Bacillus amyloliquefaciens* D747 Strain) for management of white mold in snap and dry bean. Plant Health Prog..

[84] Burkett-Cadena M., Kokalis-Burelle N., Lawrence K.S., Van Santen E., Kloepper J.W. (2008). Suppressiveness of root-knot nematodes mediated by rhizobacteria. Biol. Control.

[85] Chen X.H., Koumoutsi A., Scholz R., Eisenreich A., Schneider K., Heinemeyer I., Morgenstern B., Voss B., Hess W.R., Reva O. (2007). Comparative analysis of the complete genome sequence of the plant growth–promoting bacterium *Bacillus amyloliquefaciens* FZB42. Nat. Biotechnol..

[86] Liu X.Y., Min Y., Wang K.M., Wan Z.Y., Zhang Z.G., Cao C.X., Zhou R.H., Jiang A.B., Liu C.J., Zhang G.Y. (2014). Draft genome sequence of *Bacillus amyloliquefaciens* HB-26. Stand. Genomic Sci..

[87] Devaraj K., Aathika S., Periyasamy K., Periyaraman P.M., Palaniyandi S., Subramanian S. (2019). Production of thermostable multiple enzymes from *Bacillus amyloliquefaciens* KUB29. Nat. Prod. Res..

[88] Kalawong R., Wakayama M., Anuntalabhochai S., Wongsawad C., Sangwijit K. (2018). Comparison and characterization of purified cellulase and xylanase from *Bacillus amyloliquefaciens* CX1 and *Bacillus subtilis* B4. Chiang Mai J. Sci..

[89] Farhat-Khemakhem A., Blibech M., Boukhris I., Makni M., Chouayekh H. (2018). Assessment of the potential of the multi-enzyme producer *Bacillus amyloliquefaciens* US573 as alternative feed additive. J. Sci. Food Agric..

[90] Sewalt V., Shanahan D., Gregg L., La Marta J., Carrillo R. (2016). The Generally Recognized as Safe (GRAS) process for industrial microbial enzymes. Ind. Biotechnol..

[91] Prajapati V.S., Ray S., Narayan J., Joshi C.C., Patel K.C., Trivedi U.B., Patel R.M. (2017). Draft genome sequence of a thermostable, alkaliphilic α-amylase and protease producing *Bacillus amyloliquefaciens* strain KCP2. 3 Biotech.

[92] Meier M.J., Dodge A., Beaudette L.A. (2018). Draft genome sequence of the industrially significant bacterium *Bacillus amyloliquefaciens* NRRL 942 Matthew. Microbiol. Resour. Announc..

[93] Montor-Antonio J.J., Sachman-Ruiz B., Lozano L., del Moral S. (2015). Draft genome sequence of *Bacillus amyloliquefaciens* JJC33M, isolated from sugarcane soils in the papaloapan region, Mexico. Genome Announc..

[94] Chen L., Gu W., Xu H.-Y., Yang G.L., Shan X.F., Chen G., Wang C.F., Qian A.D. (2018). Complete genome sequence of *Bacillus velezensis* 157 isolated from *Eucommia ulmoides* with pathogenic bacteria inhibiting and lignocellulolytic enzymes production by SSF. 3 Biotech.

[95] Gong G., Kim S., Lee S.M., Woo H.M., Park T.H., Um Y. (2017). Complete genome sequence of *Bacillus* sp. 275, producing extracellular cellulolytic, xylanolytic and ligninolytic enzymes. J. Biotechnol..

[96] Hassan M. (2016). The Role of Pectin Utilization in Root Colonization and Plant Growth-Promotion by *Bacillus amyloliquefaciens* subsp. *plantarum* (Bap). Master’s Thesis.

[97] Das R., Liang Z., Li G., Mai B., An T. (2019). Genome sequence of a spore-laccase forming, BPA-degrading *Bacillus* sp. GZB isolated from an electronic-waste recycling site reveals insights into BPA degradation pathways. Arch. Microbiol..

[98] Jung J.Y., Chun B.H., Moon J.Y., Yeo S.H., Jeon C.O. (2016). Complete genome sequence of *Bacillus methylotrophicus* JJ-D34 isolated from deonjang, a korean traditional fermented soybean paste. J. Biotechnol..

[99] Yang H., Yang L., Li X., Li H., Tu Z., Wang X. (2019). Genome sequencing, purification, and biochemical characterization of a strongly fibrinolytic enzyme from *Bacillus amyloliquefaciens* Jxnuwx-1 isolated from chinese traditional douchi. J. Gen. Appl. Microbiol..

[100] Marasini D., Cornell C.R., Oyewole O., Sheaff R.J., Fakhr M.K. (2017). The whole-genome sequence of *Bacillus velezensis* strain SB1216 isolated from the great salt plains of Oklahoma reveals the presence of a novel extracellular RNase with antitumor activity. Genome Announc..

[101] Song P., Xu X., Jiang L., Zhang R., Wang J., Xu Q., Li S. (2013). Genome sequence of *Bacillus subtilis* SPZ1, an evolved strain for higher uptake rate of tributyrin. Genome Announc..

[102] Zhai L., Ren R., Meng D., Tian Q., Guan Z., Cai Y., Liao X. (2019). Comparison of aminotransferases of three *Bacillus* strains *Bacillus altitudinis* W3, *Bacillus velezensis* SYBC H47, and *Bacillus amyloliquefaciens* YP6 via genome analysis and bioinformatics. J. Appl. Genet..

[103] Wu L., Li X., Ma L., Blom J., Wu H., Gu Q., Borriss R., Gao X. (2020). The “pseudo-pathogenic” effect of plant growth-promoting bacilli on starchy plant storage organs is due to their α-amylase activity which is stimulating endogenous opportunistic pathogens. Appl. Microbiol. Biotechnol..

[104] Clatworthy A.E., Pierson E., Hung D.T. (2007). Targeting virulence: A new paradigm for antimicrobial therapy. Nat. Chem. Biol..

[105] Fazle Rabbee M., Baek K.H. (2020). Antimicrobial activities of lipopeptides and polyketides of *Bacillus velezensis* for agricultural applications. Molecules.

[106] Perlman D., Bodanszey M. (1971). Biosynthesis of peptide antibiotics. Annu. Rev. Microbiol..

[107] Hancock R.E., Chapple D.S. (1999). Peptide antibiotics. Antimicrob. Agents Chemother..

[108] Grady E.N., MacDonald J., Ho M.T., Weselowski B., McDowell T., Solomon O., Renaud J., Yuan Z.C. (2019). Characterization and complete genome analysis of the surfactin-producing, plant-protecting bacterium *Bacillus velezensis* 9D-6. BMC Microbiol..

[109] Xu B.H., Lu Y.Q., Ye Z.W., Zheng Q.W., Wei T., Lin J.F., Guo L.Q. (2018). Genomics-guided discovery and structure identification of cyclic lipopeptides from the *Bacillus siamensis* JFL15. PLoS ONE.

[110] Abdelhamid A.G., Hussein W.E., Gerst M.M., Yousef A.E. (2019). Draft genome sequence of *Bacillus velezensis* OSY-GA1, which encodes multiple antimicrobial metabolites and expresses antimicrobial activity against foodborne pathogens. Microbiol. Resour. Announc..

[111] Huffman J., Gerber R., Du L. (2010). Recent advancements in the biosynthetic mechanisms for polyketide-derived mycotoxins. Biopolymers.

[112] Weissman K.J. (2009). Introduction to polyketide biosynthesis. Methods Enzymol..

[113] Gomes E.S., Schuch V., Lemos E.G.D.M. (2013). Biotechnology of polyketides: New breath of life for the novel antibiotic genetic pathways discovery through metagenomics. Brazilian J. Microbiol..

[114] Lee H.J., Chun B.H., Jeon H.H., Kim Y.B., Lee S.H. (2017). Complete genome sequence of *Bacillus velezensis* YJ11-1-4, a strain with broadspectrum antimicrobial activity, isolated from traditional korean fermented soybean paste. Genome Announc..

[115] Zheng C.J., Lee S., Lee C.H., Kim W.G. (2007). Macrolactins O–R, glycosylated 24-membered lactones from *Bacillus* sp. AH159-1. J. Nat. Prod..

[116] Kechagia M., Basoulis D., Konstantopoulou S., Dimitriadi D., Gyftopoulou K., Skarmoutsou N., Fakiri E.M. (2013). Health benefits of probiotics: A review. ISRN Nutr..

[117] Ryu M.S., Yang H.J., Jeong S.J., Seo J.W., Ha G., Jeong S.Y., Jeong D.Y. (2018). Characteristic study and optimization of culture conditions for *Bacillus amyloliquefaciens* SRCM 100731 as probiotic resource for companion animal. Microbiol. Soc. Korea.

[118] Brown S., Dart P. (2005). Testing Hay Treated with Mould-Inhibiting, Biocontrol Inoculum.

[119] Pereira J.Q., Ritter A.C., Cibulski S., Brandelli A. (2019). Functional genome annotation depicts probiotic properties of *Bacillus velezensis* FTC01. Gene.

[120] Karlyshev A.V., Melnikov V.G., Chistyakov V.A. (2014). Draft genome sequence of *Bacillus amyloliquefaciens* B-1895. Genome Announc..

[121] Golovko G., Zipelt L., Karpenko G., Chistyakov V., Sazykina M., Kolenko M. (2008). Method for Growth of Young Azov-Chernomorskaya Royal Fish in Ponds. RU Patent.

[122] AlGburi A., Volski A., Cugini C., Walsh E.M., Chistyakov V.A., Mazanko M.S., Bren A.B., Dicks L.M.T., Chikindas M.L. (2016). Safety properties and probiotic potential of *Bacillus subtilis* KATMIRA1933 and *Bacillus amyloliquefaciens* B-1895. Adv. Microbiol..

[123] Yi Y., Zhang Z., Zhao F., Liu H., Yu L., Zha J., Wang G. (2018). Probiotic potential of *Bacillus velezensis* JW: Antimicrobial activity against fish pathogenic bacteria and immune enhancement effects on *Carassius auratus*. Fish Shellfish Immunol..

[124] Gao X.Y., Liu Y., Miao L.L., Li E.W., Sun G.X., Liu Y., Liu Z.P. (2017). Characterization and mechanism of anti-*Aeromonas salmonicida* activity of a marine probiotic strain, *Bacillus velezensis* V4. Appl. Microbiol. Biotechnol..

[125] Llario F., Romano L.A., Rodilla M., Sebastiá-Frasquet M.T., Poersch L.H. (2020). Application of *Bacillus amyloliquefaciens* as probiotic for *Litopenaeus vannamei* (Boone, 1931) cultivated in a biofloc system. Iran. J. Fish. Sci..

[126] Lin Y.S., Saputra F., Chen Y.C., Hu S.Y. (2019). Dietary administration of *Bacillus amyloliquefaciens* R8 reduces hepatic oxidative stress and enhances nutrient metabolism and immunity against *Aeromonas hydrophila* and *Streptococcus agalactiae* in zebrafish (*Danio rerio)*. Fish Shellfish Immunol..

[127] Al-Deriny S.H., Dawood M.A., Abou Zaid A.A., Wael F., Paray B.A., Van Doan H., Mohamed R.A. (2020). The synergistic effects of *Spirulina platensis* and *Bacillus amyloliquefaciens* on the growth performance, intestinal histomorphology, and immune response of Nile tilapia (*Oreochromis niloticus*). Aquacult. Repot..

[128] Chauhan A., Singh R. (2019). Probiotics in aquaculture: A promising emerging alternative approach. Symbiosis.

[129] Azrin N.A.R., Yuzine E., Ina-Salwany M.Y., Karim M. (2019). The Efficacy of Potential Probiont Bacillus amyloliquefaciens Strain L11 in Protecting Artemia Nauplii and Blue Crab Juveniles against Vibrio harveyi Infection. J. Pure Appl. Microbiol..

[130] Abatenh E., Gizaw B., Tsegaye Z., Wassie M. (2017). The role of microorganisms in bioremediation-a review. Open J. Environ. Biol..

[131] Meng D., Zhai L.X., Tian Q.P., Guan Z.B., Cai Y.J., Liao X.R. (2019). Complete genome sequence of *Bacillus amyloliquefaciens* YP6, a plant growth rhizobacterium efficiently degrading a wide range of organophosphorus pesticides. J. Integr. Agric..

