# Supplementary material for: A Review on the Biotechnological Applications of the Operational Group Bacillus amyloliquefaciens
Source: Microorganisms. 2021 Mar 17;9(3):614. doi: 10.3390/microorganisms9030614 (PMC8002464; doi:10.3390/microorganisms9030614)
Supplement: Supplementary file 1 [file microorganisms-09-00614-s001.pdf]

Table S1: Bacterial strains from the operational group *B. amyloliquefaciens*. The information source was obtained from the NCBI Entrez Genome Project database (http://www.ncbi.nlm.nih.gov/genome). Note, "NA" = not available.

Bacterial strain	Isolation source	Country	NCBI accession no.
B. amyloliquefaciens ATCC 13952	NA	NA	CP009748
B. amyloliquefaciens B425	Cereals	Netherlands	LQYP01000000
B. amyloliquefaciens CHCC26933	Soil	Japan	PQWK01000000
B. amyloliquefaciens CMW1	Japanese fermented soybean paste	Japan	BBLH01000000
B. amyloliquefaciens DSM 7	Soil and industrial amylase fermentations	Japan	FN597644
B. amyloliquefaciens HK1	Corn stalk residue compost	China	CP018902
B. amyloliquefaciens K2	Mangrove rhizophere	Thailand	MOEA01000000
B. amyloliquefaciens KCP2	Muncipal food waste	India	NMRK01000000
B. amyloliquefaciens LL3	Bibimbap	Korea	CP002634
B. amyloliquefaciens DSM 10	NA	NA	CP060710
B. amyloliquefaciens MT45	Chinese *Maotai Daqu*	China	CP011252
B. amyloliquefaciens NRRL 942	Amylase concentrates	Canada	QVEJ01000000
B. amyloliquefaciens RD7-7	Fermented soybean foods	South Korea	CP016913
B. amyloliquefaciens SRCM101266	Kochujang	South Korea	LYUG01000000
B. amyloliquefaciens SRCM101267	Food	South Korea	CP021505
B. amyloliquefaciens SRCM101294	Kochujang	South Korea	LZZO01000000
B. amyloliquefaciens TA208	NA	China	CP002627
B. amyloliquefaciens XH7	NA	China	CP002927
B. amyloliquefaciens YP6	Rhizosphere of *Lolium perenne* L. on a rock phosphorus mine	China	CP032146
B. siamensis 7551	Uncut heroin sample	Germany	NPCI01000000
B. siamensis JFL15	Gastrointestinal tract of *Trichiurus haumela*	China	LFWQ00000000
B. siamensis JJC33M	Soil	Mexico	JTJG01000000
B. siamensis KACC 16244	Crab	Thailand	GCA000262045
B. siamensis KCTC 13613	Salted thai food	Thailand	AJVF01000000
B. siamensis SCSIO 05746	Sea mud	Indian Ocean	CP025001
B. siamensis sdc15	black rice seed	India	PEKS01000000
B. siamensis SDLI1	*Scaptotrigona depilis* larva	Brazil	CP013950
B. siamensis SRCM100169	Kochujang	South Korea	LYUE01000000
B. siamensis XY18	Cured vanilla bean	China	LAGT01000000
B. velezensis 83	Mango ochard	Mexico	CP034203

Bacterial strain	Isolation source	Country	NCBI accession no.
B. velezensis 157	*Eucommia ulmoides*	China	CP022341
B. velezensis 275	Tidal flat sediment sample	South Korea	CP019626
B. velezensis 916	Soil	China	AFSU00000000
B. velezensis 7899	Uncut heroin sample	Germany	NPDH01000000
B. velezensis 10075	Chinese food lobster sauce	China	CP025939
B. velezensis 11B91	Marine	China	LPUP00000000
B. velezensis 131-4	Soil	South Korea	CP028441
B. velezensis 1B-23	Potato rhizosphere	Canada	CP033967
B. velezensis 2A-2B	Soil	Mexico	MLCV00000000
B. velezensis 3A-25B	Soil	Mexico	MLCW00000000
B. velezensis 5B6	Leaf of *Prunus avium*	South Korea	AJST01000000
B. velezensis 5RB	Lake sediment	Bulgaria	QXJL00000000
B. velezensis 7537-G2	Uncut heroin sample	Germany	NPBZ00000000
B. velezensis 7551-1	Uncut heroin sample	Germany	NPCI01000000
B. velezensis 7551-2	Uncut heroin sample	Germany	NPCJ01000000
B. velezensis 7586-G	Uncut heroin sample	Germany	NPCK00000000
B. velezensis 8-2	Soil	South Korea	CP028439
B. velezensis 9912D	Sediment sample from the Liaodong Bay of the Bohai Sea	China	CP017775
B. velezensis 9D-6	Potato rhizosphere	Canada	CP020805
B. velezensis A6	*Oryza sativa* rhizosphere	India	MSXZ01000000
B. velezensis AGVL-005	Soybean seeds	Brazil	CP024922
B. velezensis AH159-1	Mushroom	South Korea	JFBZ00000000
B. velezensis ALB65	Alfalfa silage	United States	CP029069
B. velezensis ALB69	Almond drupes	United States	CP029070
B. velezensis ANSB01E	Chicken intestine	China	CP036518
B. velezensis AP183	Cotton plant rhizosphere	United States	CP029296
B. velezensis AP194	Soil	United States	LSZL01000000
B. velezensis AP214	Soil	United States	LSZM00000000
B. velezensis AS43.3	NA	NA	CP003838
B. velezensis ATCC 12321	NA	NA	ARYD01000000
B. velezensis ATCC 19217	NA	NA	CP009749
B. velezensis B-1	Oil field	Germany	CP009684
B. velezensis B15	Grape skin	China	CP014783
B. velezensis B1895	NA	Russia	JMEG00000000
B. velezensis B25	Rhizospheric soil	Mexico	CP016285
B. velezensis B26	Switchgrass	Canada	LGAT01000000
B. velezensis B4140	Cereals	Netherlands	LQYO00000000
B. velezensis B5	Deep-sea sediment	Pacific Ocean	NRIK01000000
B. velezensis B6	Soil used for soybean agriculture	China	NEOS00000000

Bacterial strain	Isolation source	Country	NCBI accession no.
B. velezensis CAU B946	Rice rhizosphere	China	HE617159
B. velezensis B9601-Y2	Wheat rhizosphere	China	HE774679
B. velezensis Bac57	Red sea lagoons-mangrove mud	Saudi Arabia	CP033054
B. velezensis BE2	Maize rhizosphere	France	RRZG00000000
B. velezensis BH072	Honey	China	CP009938
B. velezensis BIM B-439D	Soil	Belarus	CP032144
B. velezensis Bs006	*Physalis peruviana* root	Colombia	LJAU00000000
B. velezensis BS-37	Oil from Sheng Li oil field	China	CP023414
B. velezensis Bs-916	Paddy soil	China	CP009611
B. velezensis BTLK6A	Seed of *Triticum aestivum* cv. *Kanchan*	Bangladesh	WOYD00000000
B. velezensis BTS 4	Seed in rice cultivar Rangabinni	Bangladesh	WOVK01000000
B. velezensis BUU 004	Pond sediment contaning *Penaeus monodon*	Thailand	SJCZ00000000
B. velezensis C2	Crown in *Lycopersicon esculentum* Mill.	Tunisia	NOWG01000000
B. velezensis CBMB205	Rice rhizoplane	Korea	CP014838
B. velezensis CBMC205	Rice rhizosphere	South Korea	CP011937
B. velezensis CC09	*Cinnamomum camphora* leaves	China	CP015443
B. velezensis CC178	Cucumber phyllosphere	South Korea	CP006845
B. velezensis CE2	Soil	United States	RBZX00000000
B. velezensis CFSAN034338	Agricultural soil	Canada	LYNA00000000
B. velezensis CFSAN034339	Agricultural soil	Canada	LYNB00000000
B. velezensis CFSAN034340	Agricultural soil	United States	LYNC00000000
B. velezensis CGMCC 11640	Bamboo forest soil	China	CP026610
B. velezensis CH13	Chernozem soil used for wheat agriculture	Moldova	MPHE00000000
B. velezensis CHCC26801	Water	Spain	PQWL00000000
B. velezensis CMT-6	Douchi	China	CP025341
B. velezensis CN026	Chicken feces	Belgium	CP024897
B. velezensis Co1-6	*Calendula officinalis* rhizosphere	Egypt	CVPA00000000
B. velezensis CS1.10S	Soy sauce mash	China	RCDH00000000
B. velezensis KD1	Doenjang	South Korea	CP014990
B. velezensis DC-12	Fermented soya beans	China	AMQI01000000
B. velezensis DJFZ40	Soil	China	PVRO00000000
B. velezensis DKU_NT_04	Fermented soya beans	South Korea	CP026533
B. velezensis DR-08	Soil	South Korea	CP028437
B. velezensis DSYZ	Rhizosphere	China	CP030150
B. velezensis EBL11	Rice rhizosphere	China	JCOC00000000
B. velezensis EGD-AQ14	Saline desert plant rhizosphere	India	AVQH00000000

Bacterial strain	Isolation source	Country	NCBI accession no.
B. velezensis F11	Salt lake	Algeria	MSTO00000000
B. velezensis FH17	Soil / grassland	Netherlands	RQPG00000000
B. velezensis Fito_F321	leaf of *Vitis vinifera*	Portugal	MSYT00000000
B. velezensis FKM10	Apple rhizosphere	China	LNTG00000000
B. velezensis FS001	deer feces	China	PYLR00000000
B. velezensis FTC01	Probiotic animal feed	Brazil	MAYA00000000
B. velezensis FZB42	Plant-pathogen-infested soil of a sugar beet field	Germany	CP000560
B. velezensis G341	4-year-old roots of Korean ginseng	South Korea	CP011686
B. velezensis GB03	Healthy foliage of a Douglas fir	Australia	AYTJ01000000
B. velezensis GB1	Vegetable plot used for cucumber agriculture	China	KZ155841
B. velezensis GBSW11	Soil	China	PVRP00000000
B. velezensis GD4a	NA	NA	FTNB00000000
B. velezensis GF610	Garden soil	United States	NQXV00000000
B. velezensis GFP-2	Whitespotted bamboo shark intestine	China	CP021011
B. velezensis GH1-13	Rice paddy soil	South Korea	CP019040
B. velezensis GQJK49	*Lycium barbarum* L. rhizosphere	China	CP021495
B. velezensis GR4-5	Soil	South Korea	JYGH00000000
B. velezensis GYL4	Pepper plant	Korea	CP020874
B. velezensis GZB	Electronic waste sludge of dismantling workshop	China	MTQG01000000
B. velezensis H57	Lucerne leaf	Australia	LMUC00000000
B. velezensis HB-26	Soil	China	AUWK00000000
B. velezensis HJ18-4	Fermented soybean paste	South Korea	MDCI01000000
B. velezensis Hx05	Banana rhizosphere	China	CP029473
B. velezensis IT-45	NA	NA	CP004065
B. velezensis J01	Shrimp feed	Brazil	CP023133
B. velezensis J-5	Tomato rhizosphere	China	CP018295
B. velezensis J7-1	Soil	South Korea	CP028440
B. velezensis JJ-D34	Fermented soybean product	South Korea	CP011346
B. velezensis JK	Seeds of *Oryza sativa* L., Shenliangyou 5814	China	VANQ01000000
B. velezensis JRS5	Rhizosphere of the desert plant *Rhazya stricta*	Saudi Arabia	CYHL00000000
B. velezensis JS25R	Spikelets of wheat heads	NA	CP009679
B. velezensis JT3-1	yak feces	China	CP032506
B. velezensis JTYP2	Leaves of *Echeveria laui*	China	CP020375
B. velezensis JW	Carp gastrointestinal tract	China	PPXP00000000
B. velezensis Jxnu-18	Lobster sauces	NA	OFHT00000000

Bacterial strain	Isolation source	Country	NCBI accession no.
B. velezensis Jxnuwx-1	Lobster sauces	China	LMAT00000000
B. velezensis K26	Korean fermented food	South Korea	CP023075
B. velezensis KACC 13105	Rice rhizosphere	South Korea	JTKJ00000000
B. velezensis KACC 18228	Rice endophyte	South Korea	LLZA00000000
B. velezensis KD1	Doenjang	South Korea	CP014990
B. velezensis KHG19	NA	NA	CP007242
B. velezensis L-1	Soil	China	CP023859
B. velezensis LABIM40	*Fusarium* culture contamination in plate	Brazil	CP023748
B. velezensis LB002	Fertilizer	China	CP037417
B. velezensis LDO2	Peanut root	China	CP029034
B. velezensis LFB112	Chinese herbs	China	CP006952
B. velezensis L-H15	Cucumber seedling substrate	China	CP010556
B. velezensis LK7	Plant	Malaysia	LDUN00000000
B. velezensis LM2303	Wild yak dung	China	CP018152
B. velezensis LPL-K103	Lemon samples	China	CP039380
B. velezensis L-S60	Soil	China	CP011278
B. velezensis LS69	Rice field	China	CP015911
B. velezensis Lzh-a42	Tomato rhizosphere	China	CP025308
B. velezensis M27	Cotton-waste compost	South Korea	AMPK00000000
B. velezensis M49	Ulu Slim hot spring	Malaysia	LQQW00000000
B. velezensis M75	Environment sample from cotton waste for mushroom cultivation	South Korea	CP016395
B. velezensis MBE1283	Korean traditional alcoholic beverage	South Korea	CP013727
B. velezensis MG33	Soil	Netherlands	QJJB00000000
B. velezensis MG43	Soil	Netherlands	QJJC00000000
B. velezensis MH25	Rhizosphere	China	CP034176
B. velezensis MRC 16791	Cave deposit	India	PHNH00000000
B. velezensis MRC 5958	Hot spring	India	PTTN00000000
B. velezensis NAU-B3	Wheat rhizosphere	China	HG514499
B. velezensis NB91	Human external auditory canal	China	MTID00000000
B. velezensis NBIF-001	Soil	China	CP020893
B. velezensis NBIF-003	Soil	China	LJJY00000000
B. velezensis NJAU-Z9	Field soil	China	CP022556
B. velezensis NJN-6	Rhizosphere of healthy banana plants	China	CP007165
B. velezensis NKG-1	Rare dormant volcanic soils	China	CP024203
B. velezensis NKYL29	Soil	China	JPYY00000000
B. velezensis NRRL B-41580	River Velez	Spain	LLZC00000000

Bacterial strain	Isolation source	Country	NCBI accession no.
B. velezensis NRRL B-4257	Soil	Israel	LLZB00000000
B. velezensis NWUMFk_BS10.5	Maize rhizosphere	South Africa	NITU00000000
B. velezensis NY12-2	Fermented foods	South Korea	CP033576
B. velezensis OB9	Crude oil	Canada	LGAU00000000
B. velezensis OEE1	NA	Tunisia	MZXS00000000
B. velezensis OSY-GA1	Soil	United States	CP031880
B. velezensis OSY-S3	Silage	United States	CP024706
B. velezensis P42	*Punica granatum* phylloplane	India	MSXY00000000
B. velezensis Pc3	Seawater	Antarctica	CP010406
B. velezensis PEBA20	Poplar	China	PVHM00000000
B. velezensis PG12	Apple	China	PIWI00000000
B. velezensis QST713	Commercial product SERENADE (Bayer)	France	CP025079
B. velezensis RC 218	Wheat anther	Argentina	LQCL00000000
B. velezensis RHNK22	Rhizosphere	India	LMAG00000000
B. velezensis RUPDJ	NA	NA	FTNS00000000
B. velezensis S141	Soybean rhizosphere	Thailand	AP018402
B. velezensis S3-1	Cucumber rhizosphere soil	China	CP016371
B. velezensis S499	Soil	Democratic Republic of the Congo	CP014700
B. velezensis SB1216	Soil	United States	CP015417
B. velezensis SB-9	*Vitis labrusca* x *Vitis vinifera* root	China	RRZZ00000000
B. velezensis SCDB 291	Doenjang	South Korea	CP022654
B. velezensis SCGB 1	Doenjang	South Korea	CP023320
B. velezensis SCGB 574	Doenjang	South Korea	CP023431
B. velezensis SGAir0473	Air	Singapore	CP027868
B. velezensis SK007	Soil	China	QXJQ00000000
B. velezensis SK19.001	Soil	China	AOFO00000000
B. velezensis SPZ1	NA	NA	AQGM00000000
B. velezensis SQR9	NA	NA	CP006890
B. velezensis SRCM100072	Food	South Korea	CP021888
B. velezensis SRCM100730	Kochujang	South Korea	LZZN00000000
B. velezensis SRCM100731	Kochujang	South Korea	LYUF00000000
B. velezensis SRCM101413	Food	South Korea	CP021890
B. velezensis SRCM103616	Food	South Korea	CP035410
B. velezensis SRCM103639	Food	South Korea	SDED00000000
B. velezensis SRCM103691	Food	South Korea	CP035393
B. velezensis SRCM103788	Food	South Korea	CP035399
B. velezensis SSBW-10	Slow sand biofilter	Poland	NBMO00000000

Bacterial strain	Isolation source	Country	NCBI accession no.
B. velezensis SSBW-18	Slow sand biofilter	Poland	NBMN00000000
B. velezensis SSBW-19	Slow sand biofilter	Poland	NBMM00000000
B. velezensis SSBW-2	Slow sand biofilter	Poland	NBMQ00000000
B. velezensis SSBW-8	Slow sand biofilter	Poland	NBMP00000000
B. velezensis sx01604	Soil	China	CP018007
B. velezensis SYBC H47	Honey	China	CP017747
B. velezensis T20E-257	*Solanum lycopersicum* root endosphere	South Korea	CP021976
B. velezensis TB1501	Soil	China	CP022531
B. velezensis TH16	Soil / grassland	Netherlands	RQPF00000000
B. velezensis TJ02	Soil	China	CP024797
B. velezensis TrigoCor1448	Wheat plant	Brazil	CP007244
B. velezensis UASWS BA1	Dead *Platanus* x *acerifolia*	Switzerland	AWQY00000000
B. velezensis UBA5705	Soil	China	DIHD00000000
B. velezensis UCMB5033	Cotton plant	Ukraine	NC022075
B. velezensis UCMB5036	Cotton plant	Ukraine	NC020410
B. velezensis UCMB5113	Soil	Ukraine	NC022081
B. velezensis UMAF6614	NA	NA	CP006960
B. velezensis UMAF6639	NA	NA	CP006058
B. velezensis UNC69MF	NA	NA	JQKM00000000
B. velezensis V4	Water	China	MBDV00000000
B. velezensis VCC-2003	River mud	Turkey	CP027429
B. velezensis W1	NA	China	CP028375
B. velezensis W2	Saffron field	India	JOKF00000000
B. velezensis WS-8	Soil	China	CP018200
B. velezensis X1	Soil	China	JQNZ01000018
B. velezensis XK-4-1	Cotton	China	LJDI01000010
B. velezensis Y14	Rhizosphere of peanut	China	CP017953
B. velezensis Y2	Wheat rhizosphere	NA	NC017912
B. velezensis YJ11-1-4	Soybean fermented product	South Korea	CP011347
B. velezensis ZeaDK315Endobac16	*Zea mays* endosperm	France	CP043809
B. velezensis ZF2	Cucumber plants	China	CP032154
B. velezensis ZL918	Infected bulbs of *Sagittaria sagittifolia*	China	CP021338
